# The supportive care needs of women experiencing gynaecological cancer: a Western Australian cross-sectional study

**DOI:** 10.1186/s12885-018-4812-9

**Published:** 2018-09-21

**Authors:** Natalie Williams, Georgia Griffin, Victoria Farrell, Alethea Rea, Kevin Murray, Yvonne L. Hauck

**Affiliations:** 1 Department of Nursing and Midwifery Education and Research, King Edward Memorial Hospital, Subiaco, Western Australia 6008 Australia; 2 School of Nursing, Midwifery and Paramedicine, Curtin University, Bentley, Western Australia 6102 Australia; 3 Centre for Applied Statistics, University of Western Australia, Crawley, Western Australia 6009 Australia; 4 School of Population and Global Health, University of Western Australia, Crawley, Western Australia 6009 Australia

**Keywords:** Gynaecological cancer, Supportive care, Survivorship, Cancer, Needs, Oncology

## Abstract

**Background:**

Women diagnosed with gynaecological cancer experience supportive care needs that require care provision to reduce the impact on their lives. International evidence suggests supportive care needs of women with gynaecological cancer are not being met and provision of holistic care is a priority area for action. Knowledge on gynaecological cancer supportive care needs is limited, specifically comparison of needs and cancer gynaecological subtype.

Our aim was to identify supportive care needs of Western Australian women experiencing gynaecological cancer, their satisfaction with help and explore associations between participant’s demographic characteristics and identified needs.

**Methods:**

A cross-sectional design incorporating a modified version of the Supportive Care Needs Survey – short form (SCNS-SF34) assessed 37 supportive care needs under five domains in conjunction with demographic data. Three hundred and forty three women with gynaecological cancer attending a tertiary public referral hospital completed the survey over 12 months. Statistical analysis was performed using the R environment for statistical computing. A linear regression model was fitted with factor scores for each domain and demographic characteristics as explanatory variables.

**Results:**

Three hundred and three women (83%) identified at least one moderate or high level supportive care need. The five highest ranked needs were, ‘being informed about your test results as soon as feasible’ (54.8%), ‘fears about cancer spreading’ (53.7%), ‘being treated like a person not just another case’ (51.9%), ‘being informed about cancer which is under control or diminishing (that is, remission)’ (50.7%), and ‘being adequately informed about the benefits and side-effects of treatments before you choose to have them’ (49.9%). Eight of the top ten needs were from the ‘health system and information’ domain. Associations between supportive care items and demographic variables revealed ‘cancer type’, and ‘time since completion of treatment’ had no impact on level of perceived need for any domain.

**Conclusions:**

Western Australian women with gynaecological cancer identified a high level of supportive care needs. The implementation of a supportive care screening tool is recommended to ensure needs are identified and care is patient-centred. Early identification and management of needs may help to reduce the burden on health system resources for managing ongoing needs.

**Electronic supplementary material:**

The online version of this article (10.1186/s12885-018-4812-9) contains supplementary material, which is available to authorized users.

## Background

Women diagnosed with gynaecological cancer experience not only the burden of the physical symptoms of disease and treatment, but also a multitude of psychological and social needs. These are collectively known as supportive care needs and often require the provision of services and care to treat symptoms or reduce the impact of the cancer on their lives.

Due to inconsistency around the concept “supportive care” within the literature, authors are encouraged to define their perception of the term whenever it is used [1]. One comprehensive definition of supportive care recognised is “the provision of the necessary services for those living with or affected by cancer to meet their informational, emotional, spiritual, social, or physical needs during the diagnostic, treatment or follow up phases encompassing issues of health promotion and prevention, survivorship, palliation, and bereavement” (p.378) [1]. This definition covers the wide range of holistic care needs that may present throughout the trajectory of the cancer experience. For women with gynaecological cancer, these needs may range from vaginal bleeding, pain, gastrointestinal effects, menopausal symptoms, infertility, incontinence issues and fatigue, through to fear, anxiety, matters affecting sexuality, financial concerns, relationship difficulties and spirituality challenges [2–5].

The recognition and treatment of holistic care needs is being increasingly recognised as essential to maintaining a quality of life acceptable to the individual throughout and beyond their cancer treatment [6]. Holistic care provision by health professionals for identified needs is crucial not only to promoting person-centred care but also to reduce the future burden on health care services. Provision of holistic care is acknowledged in the National Framework for Gynaecological Cancer Control and is identified as a priority area for action in the Australian cancer care setting [6].

Previous cross-sectional investigations have identified the needs of gynaecological cancer patients through a variety of survey tools. One tool, the Supportive Care Needs Survey (SCNS) [7], has been used in past studies within Australia, Canada, and the United States. For example, in 2008, the supportive care needs identified by 802 Australian women diagnosed with gynaecological cancer were reported [3]. In this cross-sectional study, women were surveyed using the short form version of the SCNS and of these gynaecological cancer survivors, 43% reported at least one moderate to high level unmet supportive care need. Fear about the cancer spreading was reported as the most common need, with other top priorities relating to both psychological and physical needs. A comparable Canadian cross-sectional study also from 2008 used a modified version of the SCNS (SCNS-gyne) to survey 103 women with gynaecological cancer to identify their supportive care needs [4]. Similar to Beesley et al.’s Australian study, fear about the cancer returning and fear about the cancer spreading were the two most commonly reported needs however, psychological needs including ‘uncertainty about the future’, ‘feelings of sadness’ and ‘anxiety’ dominated the top ten needs experienced.

More recent evidence from 2014 involved an American cross-sectional study that used an adapted version of the SCNS to survey 51 women with gynaecological cancer to determine their care needs. Consistent with previous research, fear about the cancer spreading was the top identified need equal to lack of energy/tiredness [8]. Another Canadian cross-sectional study in 2014 used the SCNS-gyne to survey 113 women with gynaecological cancer as part of a comprehensive questionnaire package to identify supportive care needs, determine their preferred service format and confirm whether higher unmet needs were associated with sociodemographic/medical characteristics [9]. While the results focussed primarily on needs in the sexuality domain, the prioritisation of psychological needs echoed the results of previous studies [3, 4, 8] with fear of the cancer returning as the most common need.

In the Australian setting another survey tool, the Cancer Survivors Unmet Needs (CaSUN) measure [10], has been used to identify the prevalence of met and unmet supportive care needs amongst women with gynaecological cancer. The CaSUN was used in 2007 to assess the quality of life, psychosocial functioning and supportive care needs of 199 Australian gynaecological cancer survivors [11]. Findings confirmed that 87.6% of women experienced a supportive care need and 52% experienced at least one unmet need. A further cross-sectional study conducted in 2011 with 45 South Australian gynaecological cancer survivors using the CaSUN measure confirmed that 55.6% of women identified at least one unmet need [12]. Most recently in 2015, another Australian study using this same CaSUN measure with 629 endometrial cancer survivors living in Queensland found 24% experienced an unmet need in the previous month with 20% ranking the need as moderate to high [13].

In addition to the CaSUN measure noted above, other survey tools have been used internationally to identify needs of women with gynaecological cancer. In Thailand, a psychosocial needs questionnaire (PNQ) was developed to assess the needs of 90 Buddhist and Muslim women with cancer of the reproductive system confirming that these women, irrespective of religion, also had psychosocial needs that were not fully met [14]. Two recent Turkish studies also investigated the needs of women with gynaecological cancer using other tools. For example, the Three Levels of Needs Questionnaire (3LNQ) measured the intensity and burden of symptoms of 134 gynaecological cancer patients revealing a high prevalence of symptoms and unmet palliative care needs [15]. The second Turkish study from 2016 also reported the learning needs of 92 women with gynaecological cancer using the Patient Learning Needs Scale (PLNS) suggesting that women required knowledge on subjects such as pain management, daily living activities, and psychological support [16].

Other international studies were found to have investigated supportive care needs relating to gynaecological cancer however they focussed on specific needs of individual gynaecological cancer sub-groups or on the needs of carers. For example, in The Netherlands, a cross-sectional study investigated the sexuality needs of 343 women with a history of cervical cancer and found 51% reported a need for information and/or professional help with their sexuality concerns [17]. Other investigated subgroups have included gynaecological cancer survivors with secondary lymphoedema, women with ovarian cancer during various phases of treatment and partners of women with gynaecological cancer [18]. Women with secondary lymphoedema identified significant levels of unmet needs in relation to information, physical symptoms and financial burden [19, 20]. For women in the ovarian cancer subgroup, identified needs included emotional needs, health system & information needs and sexuality needs [21–23]. Partners of women undergoing treatment for gynaecological cancer wanted help with psychological needs, wanted to be involved in sexual healthcare provision and had a need to be informed about the communication and coordination involving their partner’s care.

In summary, current evidence suggests that the supportive care needs of women with gynaecological cancer are not being met. In fact, a recent systematic review of the prevalence of supportive care needs amongst women with gynaecological cancer found the total burden of needs predominately related to comprehensive care and psychological concerns [18]. However, recommendations from the review suggest that current data on this topic remains limited and further quantitative studies are needed specifically to compare supportive care needs by gynaecological cancer subtype which has previously been difficult due to the use of different instruments and study designs. Therefore, to address this knowledge gap and contribute to our growing body of knowledge around supportive care needs for gynaecological cancer patients, it is essential to assess and compare the contemporary needs of this cohort to determine their needs and satisfaction with help offered to meet these needs.

## Methods

### Design and aim

The aims of this cross-sectional study were to identify supportive care needs of Western Australian (WA) women experiencing gynaecological cancer, their satisfaction with help and to explore associations between participant’s demographic characteristics and identified needs.

### Sample

Participants were recruited at the major public tertiary centre for gynaecological cancer in WA between March 2016 and April 2017 utilising a convenience sampling approach. An online sample size calculator [24], was used to calculate a minimum sample size of 340 women. This was determined based on a population size of 2880, a 50% response rate, 95% confidence level and 5% margin of error, assuming a normally distributed population. Women attending outpatient clinics during and following the completion of treatment for gynaecological cancer were approached in the waiting room by one of two nursing research assistants. Inclusion criteria for participation were having been diagnosed with a gynaecological cancer, having completed primary treatment, being over the age of 18, and English-speaking. Women were assessed for eligibility prior to being asked if they would like to participate in the study. Each survey included an information letter and instructions to complete the questionnaire. The nursing research assistant was available to answer further questions if requested. Women completed the questionnaire in the waiting room, or at home and returned it via post. Submission of a completed survey was deemed informed consent.

### Data collection tool

A cross-sectional retrospective questionnaire was chosen as the most appropriate instrument to collect a large amount of data on the supportive care needs of women who had been diagnosed with and completed treatment for a gynaecological cancer. This tool would enable descriptive and correlational analyses to be conducted to explore the associations between demographic variables and needs [25]. The Supportive Care Needs Survey short form (SCNS-SF34) was chosen for this purpose [7, 26]. SCNS-SF34 has been previously validated in an Australian cohort using pre-existing measures from the long form of the survey (SCNS-SF59) and Cronbach alpha reliability coefficients exceeded 0.86 for all domains [7].

The SCNS-SF34 includes supportive care needs items spanning five domains: psychological, health system and information, physical and daily living, patient care and support, and sexuality. We modified the Supportive Care Needs Survey – Short Form (SCNS-SF34) in order to meet our aims. Firstly, three items relevant to the needs of women with gynaecological cancer from the original SCNS Long Form (SCNS-LF59) [27] were added equalling a total of 37 items within the modified version. The original SCNS Long Form (SCNS-LF59) items are grouped under five domains with Cronbach alphas for each domain ranging between 0.87–0.96 [27]. Two items were added to the domain ‘Sexuality needs’ (‘changes in your ability to have sexual intercourse’ and ‘concerns about fulfilling your role as a partner’) and one to the domain ‘Psychological needs’ (‘accepting changes in your appearance’).

Secondly the scale for which participants were asked to select their level of need for help with each item was modified. The original SCNS-SF34 consisted of a 5-point scale separated under two headings. For each item, participants could choose either ‘not applicable’ or ‘satisfied’ under the heading ‘no need’, or ‘low’, ‘moderate’ or ‘high’ need under the heading ‘some need’. There was no option to clarify if ‘no need’ that participants were ‘not satisfied’. To capture level of need, help received and satisfaction with help, we modified the response scale into three parts. The first part included four options: ‘no need’, ‘low’, ‘moderate’ or ‘high’ need; part two sought whether help was received and part three determined if they were satisfied with the help.

The original SCNS-SF34 survey prompts participants to select their level of need for help for each item within the preceding month. As many of our potential participants attending the clinic were months or years after concluding their initial cancer treatment, our participants were asked to select their level of need since diagnosis to ensure previously experienced supportive care needs were not missed. In a separate column, participants were also asked to select if they had received help with that need and further, if they were satisfied with the level of help they received, both using a yes/no format. Demographic data was also collected and included continuous variables such as participant age, and length of time since completion of treatment as well as categorical data, for example cancer type, types of treatment received, relationship status, level of partner support, whether they had participated in sexual activity since diagnosis, and whether they had been diagnosed with a recurrence.

This quantitative data was collected as part of a mixed methods approach to investigating the supportive care needs of WA women with gynaecological cancer. Qualitative data was also collected for analysis including short answer written responses on the survey and telephone interviews and the results will be published elsewhere.

### Statistical analysis

The data was analysed using the R environment for statistical computing [28]. The internal reliability of the questionnaire was assessed using exploratory factor analysis, in particular the principal axis method of factor extraction, to examine the domains of need as subsets and assess the quality of individual items in the questionnaire. Eigenvalues, scree plots and hierarchical clustering of variables were investigated to determine the appropriate number of factors to report. Internal consistency of the modified tool was assessed using Cronbach’s alpha. The overall Cronbach’s alpha was 0.96 which indicates a reliable instrument.

In relation to the survey tool, factor loadings for each item with the five domains (F1-F5) were calculated and are shown in Table 1. Factor loadings and percentage of variance explained are provided for each factor, as well as the total percentage of variance explained by the model. A factor loading greater than 0.40 on the hypothesized factor was set as evidence and the lowest factor loading for any item in this survey was calculated at 0.50 indicating reliability of the tool, the domains and individual items.

**Table 1:** Factor Analysis of Survey Tool

Supportive Care Need	F1	F2	F3	F4	F5
Health System and Information Needs					
Being informed about your test results as soon as feasible	0.89				
Being informed about cancer which is under control or diminishing (that is, remission)	0.85				
Being adequately informed about the benefits and side-effects of treatments before you choose to have them	0.84				
Being given explanations of those tests for which you would like explanations	0.84				
Being treated in a hospital or clinic that is as physically pleasant as possible	0.83				
Being treated like a person not just another case	0.82				
Being given written information about the important aspects of your care	0.78				
Being given information (written, diagrams, drawings) about aspects of managing your illness and side-effects at home	0.78				
Being informed about things you can do to help yourself to get well	0.78				
Having one member of hospital staff with whom you can talk about all aspects of your condition, treatment and follow-up	0.73				
Having access to professional counselling (e.g., psychologist, social worker, counsellor, nurse specialist) if you, family or friends need it	0.59				
Psychological Needs					
Feelings of sadness		0.78			
Feelings about death or dying		0.77			
Uncertainty about the future		0.73			
Fears about the cancer spreading		0.71			
Worry that the results of the treatment are beyond your control		0.70			
Feeling down or depressed		0.69			
Anxiety		0.69			
Keeping a positive outlook		0.66			
Learning to feel in control of the situation		0.61			
Concerns about the worries of those close to you		0.59			
Accepting changes in your appearance		0.51			
Sexuality Needs					
Changes in your sexual relationships			0.92		
Changes in sexual feelings			0.90		
Changes in your ability to have sexual intercourse			0.80		
Concerns about fulfilling your role as a partner			0.64		
To be given information about sexual relationships			0.60		
Physical and Daily Living Needs					
Not being able to do the things you used to do				0.82	
Work around the home				0.70	
Feeling unwell a lot of the time				0.67	
Pain				0.58	
Lack of energy/tiredness				0.50	
Patient Care and Support Needs					
More choice about which hospital you attend					0.65
More choice about which cancer specialists you see					0.57
*Total percentage of variation*	*22%*	*18%*	*10%*	*8%*	*3%*
*Total percentage of factor model*	*22%*	*40%*	*50%*	*58%*	*61%*

For each domain, a linear regression model was fitted with the factor scores from the exploratory factor analysis as the response, and the demographic variables as explanatory variables. Model selection was carried out where variables significant at the 5% level were retained for the final model. Coefficient estimates, standard errors and *p*-values are provided for each model.

### Ethical considerations

Ethical approval to conduct the study was granted from the Human Research Ethics Committee of the tertiary hospital (Ethics Approval Number 2016030QK). Data collection and storage adhered to the principles set by the Australian National Health and Medical Research Council [29].

## Results

A total of 712 women were approached and, based on meeting the inclusion criteria, 343 women completed the survey. Demographic and disease characteristics are presented in Table 2. Participant age ranged from 18 to 99 years, with 82% of participants between 35 and 75 years. Most participants were in a relationship and living together (55.1%), sexually active (58.9%) and had quite or extremely supportive partners (58%). The most common cancer type was cervical, affecting 32.7% of participants, followed by ovarian and uterine/endometrial cancers, each affecting 26.2% of participants. The majority of survey participants were treated with surgery (80.8%) with or without another treatment modality such as chemotherapy or radiation. 9.6% of respondents had experienced a recurrence. Participants completed the survey 0–264 months since completion of treatment with a median time of 17 months.

**Table 2:** Participant Demographics (*N* = 343)

Demographic	Frequency (*n*)	Percentage (%)
Age (years)		
Under 35	38	11.1
35 to 55	124	36.2
55 to 75	157	45.8
Over 75	24	7.0
Relationship Status		
Not in a relationship	122	35.6
In a relationship - not living together	28	8.2
In a relationship - living together	189	55.1
*Participated in Sexual Activity	202	58.9
^#^Partner Support		
No support or a little supportive	19	8.1
Somewhat supportive	18	7.6
Quite supportive	62	26.3
Extremely supportive	137	58.0
Type of Cancer		
Ovarian	90	26.2
Uterine/Endometrial	90	26.2
Cervical	112	32.7
Vulval	29	8.5
Other	20	5.8
Received Surgery	277	80.8
Received Chemotherapy	130	37.9
Received Radiation	118	34.4
Cancer Reoccurred	33	9.6

*of *n* = 340; ^#^of *n* = 236 who had a partner

### Supportive care needs and satisfaction with help

Results indicating the level of need for each supportive care item are presented in Table 3 listed under the five domains. These results are presented under the four levels of need, but in order to calculate the supportive care need reported at the highest levels of need, further analysis was conducted to combine the moderate and high responses. In total 303 (83%) out of the 343 women surveyed, identified at least one moderate or high level need across the 37 items. The top supportive care needs for each domain were: Health system and information needs ‘being informed about your test results as soon as feasible’ (55%); Psychological needs ‘fears about the cancer spreading’ (54%); Sexuality needs ‘changes in your ability to have sexual intercourse’ (33%) and ‘changes in sexual feelings’ (33%); Physical and daily living needs ‘lack of energy/tiredness’ (44%), and; Patient care and support needs ‘hospital staff acknowledging, and showing sensitivity to, your feelings and emotional needs’ (47%).

**Table 3:** Level of need for supportive care under five domains

Supportive Care Need	Level of need for help *n* (%)			
	No need	Low	Mod	High
Health System and Information Needs Domain				
Being given written information about the important aspects of your care	124 (36)	74 (22)	68 (20)	77 (22)
Being given information (written diagrams, drawings) about aspects of managing your illness and side-effects at home	125 (36)	78 (23)	65 (19)	75 (22)
Being given explanation of those tests for which you would like explanations	109 (32)	75 (22)	76 (22)	83 (24)
Being adequately informed about the benefits and side-effects of treatments before you choose to have them	115 (34)	57 (17)	69 (20)	102 (30)
Being informed about your test results as soon as feasible	100 (29)	55 (16)	61 (18)	127 (37)
Being informed about cancer which is under control or diminishing (that is, remission)	109 (32)	60 (17)	61 (18)	113 (33)
Being informed about things you can do to help yourself to get well	125 (36)	53 (15)	76 (22)	89 (26)
Having access to professional counselling (e.g., psychologist, social worker, counsellor, nurse specialist) if you, family or friends need it	147 (43)	59 (17)	61 (18)	76 (22)
Being treated like a person not just another case	122 (36)	43 (13)	68 (20)	110 (32)
Being treated in a hospital or clinic that is as physically pleasant as possible	118 (34)	58 (17)	70 (20)	97 (28)
Having one member of hospital staff with whom you can talk about all aspects of your condition, treatment and follow-up	132 (38)	57 (17)	66 (19)	88 (26)
Psychological Needs Domain				
Anxiety	121 (35)	77 (22)	99 (29)	46 (13)
Feeling down or depressed	129 (38)	91 (27)	92 (27)	31 (9)
Feelings of sadness	137 (40)	97 (28)	77 (22)	32 (9)
Fears about the cancer spreading	72 (21)	87 (25)	98 (29)	86 (25)
Worry that the results of treatment are beyond your control	125 (36)	92 (27)	79 (23)	47 (14)
Accepting changes in your appearance	186 (54)	76 (22)	53 (15)	28 (8)
Uncertainty about the future	118 (34)	84 (24)	88 (26)	53 (15)
Learning to feel in control of your situation	157 (46)	81 (24)	72 (21)	33 (10)
Keeping a positive outlook	164 (48)	63 (18)	73 (21)	43 (13)
Feelings about death or dying	169 (49)	84 (24)	57 (17)	33 (10)
Concerns about the worries of those close to you	126 (37)	72 (21)	90 (26)	55 (16)
Sexuality Needs Domain				
Changes in your ability to have sexual intercourse	189 (55)	43 (13)	54 (16)	57 (17)
Changes in sexual feelings	178 (52)	53 (15)	61 (18)	51 (15)
Changes in your sexual relationships	198 (58)	46 (13)	55 (16)	44 (13)
To be given information about sexual relationships	211 (62)	50 (15)	37 (11)	45 (13)
Concerns about fulfilling your role as a partner	199 (58)	49 (14)	53 (15)	42 (12)
Physical and Daily Living Needs Domain				
Pain	168 (49)	86 (25)	50 (15)	39 (11)
Lack of energy/tiredness	122 (36)	69 (20)	92 (27)	60 (17)
Feeling unwell a lot of the time	176 (51)	79 (23)	53 (15)	35 (10)
Work around the house	196 (57)	63 (18)	60 (17)	24 (7)
Not being able to do the things you used to do	166 (48)	78 (23)	60 (17)	39 (11)
Patient Care and Support Needs Domain				
More choice about which cancer specialists you see	208 (61)	66 (19)	38 (11)	31 (9)
More choice about which hospital you attend	213 (62)	60 (17)	42 (12)	28 (8)
Reassurance by health professionals that the way you feel is normal	155 (45)	69 (20)	70 (20)	49 (14)
Hospital staff attending to your physical needs	155 (45)	68 (20)	61 (18)	59 (17)
Hospital staff acknowledging, and showing sensitivity to, your feelings and emotional needs	124 (36)	59 (17)	65 (19)	95 (28)

The results of the combined moderate and high levels of need were ranked to determine the ten most prevalent supportive care needs requiring help across all domains (Table 4). The five highest ranked supportive care needs were, ‘being informed about your test results as soon as feasible’ (54.8%), ‘fears about cancer spreading’ (53.7%), ‘being treated like a person not just another case’ (51.9%), ‘being informed about cancer which is under control or diminishing (that is, remission)’ (50.7%), and ‘being adequately informed about the benefits and side-effects of treatments before you choose to have them’ (49.9%). The top ten needs of participants had at least 44% of respondents reporting those needs. Eight of the top ten supportive care needs were from the ‘health system and information needs’ domain. One was from the ‘psychological needs’ domain and one was from the ‘patient care and support needs’ domain.

**Table 4:** Highest ranked ten supportive care needs identified at Moderate or High Level

Priority	Issue	No need	Low	Mod	High	Mod+High (*n*)	Mod+High(%)
1	Being informed about your test results as soon as feasible	100	55	61	127	188	54.81
2	Fears about the cancer spreading	72	87	98	86	184	53.64
3	Being treated like a person not just another case	122	43	68	110	178	51.89
4	Being informed about cancer which is under control or diminishing (that is, remission)	109	60	61	113	174	50.73
5	Being adequately informed about the benefits and side-effects of treatments before you choose to have them	115	57	69	102	171	49.85
6	Being treated in a hospital or clinic that is as physically pleasant as possible	118	58	70	97	167	48.69
7	Being informed about things you can do to help yourself to get well	125	53	76	89	165	48.10
8	Hospital staff acknowledging, and showing sensitivity to, your feelings and emotional needs	124	59	65	95	160	46.65
9	Being given explanation of those tests for which you would like explanations	109	75	76	83	159	46.35
10	Having one member of hospital staff with whom you can talk about all aspects of your condition, treatment and follow-up	132	57	66	88	154	44.90

Satisfaction with help received for particular supportive care needs are presented in Table 5. The needs with the highest level of satisfaction around help received included: ‘concerns about fulfilling your role as a partner’ (87%) under the ‘sexuality needs’ domain and ‘work around the house’ under the ‘physical and daily living needs’ domain (86%). The levels of satisfaction for help with care for needs under the ‘health system and information needs’ domain ranged from 29 to 53%.

**Table 5:** Summary of satisfaction with help received for supportive care needs

Satisfaction	Yes *n* (%)	No *n* (%)	Missing
Health System and Information Needs Domain			
Being given written information about the important aspects of your care	98 (37)	164 (63)	78
Being given information (written diagrams, drawings) about aspects of managing your illness and side-effects at home	99 (38)	162 (62)	81
Being given explanation of those tests for which you would like explanations	92 (34)	176 (66)	74
Being adequately informed about the benefits and side-effects of treatments before you choose to have them	83 (31)	183 (69)	76
Being informed about your test results as soon as feasible	79 (29)	193 (71)	69
Being informed about cancer which is under control or diminishing (that is, remission)	84 (32)	179 (68)	78
Being informed about things you can do to help yourself to get well	119 (47)	136 (53)	85
Having access to professional counselling (e.g., psychologist, social worker, counsellor, nurse specialist) if you, family or friends need it	137 (53)	121 (47)	84
To be given information about sexual relationships	147 (65)	79 (35)	117
Being treated like a person not just another case	88 (35)	164 (65)	91
Being treated in a hospital or clinic that is as physically pleasant as possible	76 (29)	184 (71)	82
Having one member of hospital staff with whom you can talk about all aspects of your condition, treatment and follow-up	113 (44)	141 (56)	88
Psychological Needs Domain			
Anxiety	190 (75)	65 (25)	88
Feeling down or depressed	201 (79)	52 (21)	90
Feelings of sadness	185 (76)	58 (24)	100
Fears about the cancer spreading	165 (61)	104 (39)	74
Worry that the results of treatment are beyond your control	178 (70)	75 (30)	90
Accepting changes in your appearance	188 (81)	44 (19)	111
Uncertainty about the future	182 (74)	63 (26)	97
Learning to fell in control of your situations	191 (77)	56 (23)	96
Keeping a positive outlook	177 (74)	63 (26)	103
Feelings about death or dying	194 (83)	40 (17)	109
Concerns about the worries of those close to you	201 (83)	42 (17)	100
Sexuality Needs Domain			
Changes in your ability to have sexual intercourse	196 (80)	50 (20)	96
Changes in sexual feelings	186 (79)	49 (21)	107
Changes in your sexual relationships	190 (83)	40 (17)	112
Concerns about fulfilling your role as a partner	201 (87)	29 (13)	113
To be given information about sexual relationships	147 (65)	79 (35)	117
Physical and Daily Living Needs Domain			
Pain	157 (57)	120 (43)	66
Lack of energy/tiredness	211 (80)	52 (20)	80
Feeling unwell a lot of the time	193 (77)	58 (23)	92
Work around the house	204 (86)	33 (14)	105
Not being able to do the things you used to do	198 (81)	45 (19)	100
Patient Care and Support Needs Domain			
More choice about which cancer specialists you see	183 (81)	44 (19)	116
More choice about which hospital you attend	180 (81)	42 (19)	121
Reassurance by health professionals that the way you feel is normal	150 (61)	96 (39)	96
Hospital staff attending to your physical needs	96 (37)	164 (63)	83
Hospital staff acknowledging, and showing sensitivity to, your feelings and emotional needs	95 (36)	166 (64)	80

### Associations between demographic data and supportive care needs

Associations between the perceived need for supportive care items in each domain using factor scores identified from the factor analysis, and the demographic variables of the participants are shown in Table 6. An Additional file 1: Table S1 has been provided with the univariate results for transparency and to clarify which variables were included in this multivariable analysis. Notably, the variables ‘cancer type’, and ‘time since completion of treatment’ had no impact on any level of perceived need for any of the supportive care need domains in this population of women.

**Table 6:** Multivariate results from a linear regression analysis of perceived need

	Health system and information needs		Psychological needs		Sexuality needs		Physical and daily living needs		Patient care and support needs	
	Estimate (Std Error)	*P*-value	Estimate (Std Error)	*P*-value	Estimate (Std Error)	*P*-value	Estimate (Std Error)	*P*-value	Estimate (Std Error)	*P*-value
(Intercept)	0.178 (0.138)	0.197	0.065 (0.097)	0.507	−0.254 (0.261)	0.332	0.068 (0.135)	0.614	−0.038 (0.095)	0.645
Age group										
Under 35	0.029 (0.179)	0.873	0.154 (0.179)	0.393	−0.219 (0.163)	0.181	0.114 (0.175)	0.515		
55 up to 75	−0.432 (0.122)	< 0.001	−0.422 (0.117)	< 0.001	− 0.632 (0.106)	< 0.001	−0.313 (0.119)	0.009		
75 and over	−0.643 (0.226)	0.005	−0.607 (0.214)	0.005	−0.905 (0.199)	< 0.001	−0.173 (0.221)	0.433		
*Overall*		*< 0.001*		*< 0.001*		*< 0.001*		*0.024*		
Relationship status										
In a relationship - not living together									0.781 (0.209)	< 0.001
In a relationship - living together									−0.193 (0.127)	0.121
*Overall*										*0.001*
Sexually active	−0.24 (0.116)	0.04					−0.393 (0.114)	0.001	−0.393 (0.121)	0.006
Partner support										
Somewhat supportive					0.295 (0.288)	0.306				
Quite supportive					0.421 (0.228)	0.066				
Extremely supportive					0.414 (0.213)	0.053				
Not applicable					−0.224 (0.218)	0.305				
*Overall*						*< 0.001*				
Cancer has reoccurred			0.41 (0.176)	0.021					0.762 (0.177)	< 0.001
Had surgery					0.298 (0.141)	0.035				
Had chemotherapy	0.282 (0.111)	0.012			0.225 (0.102)	0.029	0.416 (0.108)	< 0.001		
Had radiation	0.273 (0.114)	0.017	0.349 (0.112)	0.002	0.346 (0.117)	0.003	0.43 (0.111)	< 0.001		

Significant variables only. The reference category for age is ‘35 up to 55 years old’. The reference category for relationship status is ‘not in a relationship’ and ‘little or no support’ for partner support

Of the women surveyed, younger age (*p* < 0.001), sexual inactivity (*p* = 0.040), having chemotherapy (*p* = 0.012), and having radiation (*p* = 0.017), were associated with an increased perceived need of supportive care for items in the ‘health system and information needs’ domain. For psychological needs, younger age (*p* < 0.001), recurrence (*p* = 0.021) and radiation (*p* = 0.002) were associated with an increased level of need. In the sexuality needs domain, younger age (*p* < 0.001), having a quite or extremely supportive partner (*p* < 0.001), surgery (*p* = 0.035), chemotherapy (*p* = 0.029), radiation (*p* = 0.003) were all associated with an increased perceived level of need. For physical and daily living needs, not being middle-aged (55–75 years) (*p* = 0.009) (as a subgroup of age), sexual inactivity (*p* = 0.001), chemotherapy (*p* < 0.001) and radiation (*p* < 0.001) were associated with increased perceived level of need. Finally, for patient care needs, being in a relationship while not living together, when compared to not being in a relationship (*p* = 0.001), sexual inactivity (*p* = 0.006) and recurrence (*p* < 0.001) were all associated with an increased level of supportive care need.

## Discussion

Three hundred and forty three WA women with a gynaecological cancer participated in this cross-sectional study and 83% identified at least one moderate or high level supportive care need. The highest ranked needs were: ‘being informed about your test results as soon as feasible’; ‘fears about cancer spreading’; and ‘being treated like a person not just another case’. Eight of the top ten needs were from the ‘health system and information’ domain. The levels of satisfaction for help with care needs under the ‘health system and information needs’ domain were all below 50% aside from ‘having access to professional counselling’. Associations between supportive care items and demographic variables revealed ‘cancer type’, and ‘time since completion of treatment’ had no impact on any level of perceived need. Cervical cancer was the most common cancer reported followed by ovarian and uterine/endometrial cancers. This distribution did not reflect the 2014 WA profile for gynaecological cancer diagnosis with uterine cancer cited as the most reported gynaecological cancer, second was ovarian cancer and third was cervical cancer with 118 women diagnosed [30].

Our investigation was the first of its kind to be conducted with gynaecological cancer patients in WA and has provided a rich source of evidence relating to the supportive care needs of this cohort of women. The provision of person-centred care is being increasingly highlighted as a priority in the gynaecological cancer setting [6]. Cancer patients now expect to be well informed about their disease and care to assist them in making informed decisions about treatment options [31]. In our contemporary world where seeking information is assisted by media, such as the internet, expectations on health care are changing in order to meet patients’ holistic needs [32–35]. In order to ensure that health professionals are meeting the holistic care needs of cancer patients, it is essential to identify what those needs are and evaluate whether care delivery meets those expectations. It is recognised that international evidence in relation to the supportive care needs of women with gynaecological cancer is limited [18], particularly in the Australian context. This discussion will focus on key concepts reflected in our results and are compared to existing evidence to inform cancer care professionals on contemporary supportive care needs of Australian women with gynaecological cancer. The topics to be discussed include information needs, satisfaction and evidence for individualised assessment of supportive care needs.

Our study results indicated an overall high prevalence of supportive care needs amongst a cohort of WA women with gynaecological cancer with 83% of women identifying at least one moderate or high level need. The top ranked moderate to high level needs were predominantly from the domain ‘health system and information needs’. The current process for gynaecologic cancer care in WA can involve multi-centred treatment if the women requires surgical, chemotherapy and radiation services. The study site is a public tertiary referral centre and state wide service that provides specialist gynaecological oncology care for women that also includes palliative care and perinatal loss services. Initial referral and assessment is conducted at the study site. Surgical treatment is primarily performed at this centre by gynaecological oncologists with a multidisciplinary approach unless medical conditions and complications indicate that an Intensive Care Unit may be required post-surgery. However, chemotherapy and radiation treatment are provided by other centres. Women can then return to this tertiary referral centre for ongoing follow up. This potential disruption to continuity of care across clinical settings during the cancer journey may explain why ‘health system and information needs’ were ranked highly. The importance of having continuity of care across the patient journey was acknowledged by colorectal cancer survivors in Canada who confirmed that having continuity contributed to higher satisfaction with follow up care [36]. The reality for the majority of these cancer survivors was having multiple providers which presents challenges to health professionals attempting to recognise and consistently meet individual patient needs and expectations.

The top identified need at any level, amongst all women was ‘Fears about the cancer spreading’ from the psychological domain and echoed the results of previous studies [3, 8]. However, amongst these Western Australian women, there was a predominance of ‘Health system and information needs’ with items from this domain representing 8 of the top 10 needs identified. Items included ‘Being informed about your test results as soon as feasible’, ‘Being treated like a person not just another case’, ‘Being informed about cancer which is under control or diminishing’, and ‘Being informed about things you can do to help yourself to get well’. This differed from the results of earlier studies where top 10 previously identified needs were most often from the psychological domain including items such as ‘Concerns about the worries of those close to you’, ‘Uncertainty about the future’, ‘Feelings of sadness’ and ‘Feeling down or depressed’ [3, 4, 8, 9].

The prioritisation of informational needs identified in this Western Australian cohort are supported by the results of a recent Turkish cross-sectional study which investigated the learning needs of gynaecologic cancer survivors [16]. It was found that 71% (*n* = 65) of the 92 participants required information on topics such as coping with pain, daily living activities and psychological support [16]. More research is needed to establish whether the predominance of health system and information needs in the Western Australian cohort can be explained by reasons such as changing expectations for information provision due to ease of access to internet based information or an institutional based deficit caused by other possible reasons, such as inadequate clinical resources or inadequate staff education.

No further evidence could be found specifically on the prevalence of informational needs of gynaecological cancer patients and whether this need has changed over time or been influenced by modern changes in information seeking practices. Gagnon and Sabus [33] state that information seeking by health consumers and communication using online platforms such as social media is “here to stay” (p.413). They suggest the use of these digital technologies is constantly evolving and health care professionals need to adapt to meet the expectations and needs of consumers. An Australian cross-sectional study of 325 Australian breast cancer patients, replicated from 10 years previously, investigated information sources used by these women [37]. It was found that 70% (*n* = 229) of breast cancer patients used the internet for information, a significantly higher percentage compared with the previous study where only 4% (*n* = 9) identified using this resource. This result reflected a change in information seeking practices over time amongst this group of women. Again, further research is needed to determine if a similar change is occurring amongst women with gynaecological cancer.

The pre-dominance of health system and informational needs amongst these Western Australian women also differed from the results of a previous cross-sectional study investigating the supportive care needs of a wider cohort of 786 Western Australian cancer patients [38]. In this study, the SCNS long form tool (SCNS-LF59) was used and thirty four women with gynaecological cancer represented 4.4% of the sample. The five main cancer groups represented amongst the cohort were breast, prostate, colorectal, melanoma of skin and lymphoma. The top ten needs identified at any level for the total cohort were all from the domains ‘psychological’ and ‘physical and daily living’ except for number 10, which was ‘changes in sexual feelings’ from the sexuality domain, with no representation of needs from the ‘health systems and information’ domains as found in our study. The top identified need of the total cohort was “Fears about the cancer returning”, a need that did not feature in the top ten identified needs from our Western Australian cohort of women with gynaecological cancer. Within White et al.’s [38] cohort, the needs of women with gynaecological cancer only were not reported, however the needs of other certain disease groups were analysed individually and some groups were found to have differing priorities for supportive care needs, supporting the need for individualised assessment.

Results from our Western Australian study confirm that while some of the demographic variables collected, such as age, relationship status, level of partner support and type of treatment, showed significant associations with certain domains of supportive care needs, there were two variables that did not display statistical significance. These were ‘time since treatment’ and ‘type of cancer’. This was a clinically important finding given the limited data previously available for the needs of women with specific gynaecological cancer subtypes [18] and supports the implementation of individualised supportive care need assessments. It was noted from this Western Australian cohort of women that it was not possible for clinicians to pre-determine supportive care needs based on the type of gynaecological cancer diagnosed. While other demographic data may indicate a potential need in some areas, pre-determination of needs could be a risky practice, would not follow the principles of patient-centred holistic care and may result in missing the identification of needs in the absence of a formalised assessment process.

This notion of individually assessing patients for their supportive care needs is supported by a recent systematic review investigating the patterns and predictors of unmet supportive care needs in cancer patients. Okediji, Salako and Fatiregun [39] similarly found there are associations with sociodemographic and clinical factors which influence the pattern of unmet needs. They suggest that addressing these deficits is essential to achieving optimal cancer care and satisfaction but in order to do that, there is a need to develop a consistent process to identify the needs of patients using a standardised instrument in clinical practice.

Cancer Australia [6] support the delivery of comprehensive supportive care screening for women with gynaecological cancer and recommend that assessment should be performed at key points along the cancer care pathway. Formal screening was previously utilised in a research study on Western Australian women with gynaecological cancer, using the Distress Thermometer and Problem List Tool [40]. However, at the time when the data was collected for our study, the screening was no longer in use due to the tool not being implemented into clinical practice after the conclusion of the study. Interestingly, O’Connor et al. [39] found that during the tool’s use, staff perceived benefits were to validate patients’ concerns and issues and use as an icebreaker to aid communication. They also determined that using the tool assisted in enhancing their practice, offered a more holistic approach to care and avoided missing important issues. Identified challenges included time to administer the tool and choosing the best time for it to be administered.

### Limitations

Study limitations must be considered when reflecting on our results. Firstly, the demographic data collected was patient-reported and therefore the ‘type of cancer’ patients identified relied on their accurate understanding and recall of the cancer diagnosis. Given the high level of informational needs reported by these patients, it is possible this may not have always been an accurate representation of the true medical diagnosis. Secondly, no demographic data on socio-economic indexes (SEIFA) was collected for this cohort of patients. In retrospect, collecting this information would have been useful in assisting readers to determine the generalisability of results to their own societal context. Finally, the survey was completed by patients at one point in time during their cancer experience. Many factors may have influenced their responses when completing the survey such as their level of stress or anxiety, the length of waiting time, or their relationship and satisfaction with their current health care provider.

## Conclusion

The assessment of the supportive care needs of Western Australian women with gynaecological cancer highlighted important points that can be addressed to improve the care provided. Although cancer type and time since completion of treatment were not associated with particular needs, these women have a high level of supportive care needs, particularly in the ‘Health system and information needs’ domain. It is not possible for health care professionals to pre-determine what these needs might be based on the demographic variables of type of cancer or time since treatment. The implementation of a comprehensive but succinct supportive care screening tool is recommended for ensuring that supportive care needs are identified and care is patient-centred and individualised for each woman. Early identification and management of needs may then help to reduce the burden on health system resources for managing ongoing needs.

## Additional file

Additional file 1: **Table S1.** Univariate results from a linear regression analysis of perceived need. (DOCX 20 kb)
