# Supplementary material for: The supportive care needs of women experiencing gynaecological cancer: a Western Australian cross-sectional study
Source: BMC Cancer. 2018 Sep 21;18:912. doi: 10.1186/s12885-018-4812-9 (PMC6151067; doi:10.1186/s12885-018-4812-9)
Supplement: Supplementary file 1 — Table S1. Univariate results from a linear regression analysis of perceived need. (DOCX 20 kb) [file 12885_2018_4812_MOESM1_ESM.docx]

Table S1. Univariate results from a linear regression analysis of perceived need

|  | Health system and information needs | | Psychological needs | | Sexuality needs | | Physical and daily living needs | | Patient care and support needs | |
| --- | --- | --- | --- | --- | --- | --- | --- | --- | --- | --- |
|  | Estimate  (Std Error) | P-value | Estimate  (Std Error) | P-value | Estimate  (Std Error) | P-value | Estimate  (Std Error) | P-value | Estimate  (Std Error) | P-value |
| Age group |  |  |  |  |  |  |  |  |  |  |
| Under 35 | -0.073 (0.183) | 0.693 | 0.079 (0.181) | 0.661 | -0.360 (0.171) | 0.037 | -0.041 (0.186) | 0.825 | -0.262 (0.186) | 0.160 |
| 55 up to 75 | -0.360 (0.119) | 0.003 | -0.421 (0.117) | <0.001 | -0.760 (0.111) | <0.001 | -0.195 (0.120) | 0.105 | -0.199 (0.223) | 0.374 |
| 75 and over | -0.519 (0.220) | 0.019 | -0.606 (0.218) | 0.006 | -1.176 (0.206) | <0.001 | 0.037 (0.223) | 0.870 | -0.262 (0.186) | 0.160 |
| *Overall* |  | 0.007 |  | <0.001 |  | <0.001 |  | 0.361 |  | 0.492 |
| Relationship status |  |  |  |  |  |  |  |  |  |  |
| In a relationship - not living together | 0.455 (0.209) | 0.030 | 0.174 (0.210) | 0.409 | 0.638 (0.20) | 0.002 | 0.330 (0.209) | 0.116 | 0.576 (0.207) | 0.006 |
| In a relationship - living together | 0.009 (0.116) | 0.939 | 0.014 (0.117) | 0.905 | 0.659 (0.111) | <0.001 | -0.083 (0.116) | 0.473 | 0.053 (0.115) | 0.645 |
| *Overall* |  | 0.075 |  | 0.701 |  | <0.001 |  | 0.121 |  | 0.019 |
| Sexually active | -0.043 (0.110) | 0.693 | 0.034 (0.111) | 0.759 | 0.669 (0.103) | <0.001 | -0.282 (0.110) | 0.011 | -0.217 (0.110) | 0.049 |
| Partner support |  |  |  |  |  |  |  |  |  |  |
| Somewhat supportive | -0.409 (0.328) | 0.214 | 0.081 (0.329) | 0.806 | 0.361 (0.307) | 0.241 | 0.115 (0.330) | 0.726 | 0.452 (0.330) | 0.172 |
| Quite supportive | -0.232 (0.262) | 0.375 | 0.049 (0.262) | 0.852 | 0.445 (0.245) | 0.070 | -0.292 (0.263) | 0.267 | 0.218 (0.263) | 0.408 |
| Extremely supportive | -0.334 (0.244) | 0.172 | -0.050 (0.245) | 0.839 | 0.447 (0.229) | 0.052 | -0.075 (0.246) | 0.762 | 0.053 (0.245) | 0.831 |
| Not applicable | -0.401 (0.249) | 0.108 | -0.141 (0.250) | 0.571 | -0.377 (0.233) | 0.107 | -0.129 (0.250) | 0.607 | -0.009 (0.250) | 0.970 |
| *Overall* |  | 0.512 |  | 0.766 |  | <0.001 |  | 0.498 |  | 0.321 |
| Type of cancer |  |  |  |  |  |  |  |  |  |  |
| Uterine/Endometrial | -0.283 (0.148) | 0.056 | -0.029 (0.147) | 0.844 | -0.281 (0.146) | 0.056 | -0.216 (0.148) | 0.145 | -0.098 (0.149) | 0.512 |
| Cervical | 0.205 (0.140) | 0.144 | 0.406 (0.139) | 0.004 | 0.270 (0.139) | 0.053 | 0.261 (0.140) | 0.064 | 0.030 (0.142) | 0.830 |
| Vulva | -0.165 (0.212) | 0.437 | -0.122(0.210) | 0.559 | -0.117 (0.210) | 0.579 | -0.001 (0.212) | 0.995 | 0.078 (0.213) | 0.715 |
| Other | -0.103 (0.245) | 0.674 | 0.276 (0.243) | 0.257 | -0.044 (0.243) | 0.856 | 0.189 (0.245) | 0.443 | 0.030 (0.247) | 0.905 |
| Overall |  | 0.013 |  | 0.005 |  | 0.003 |  | 0.017 |  | 0.887 |
| Time since treatment | 0.002 (0.002) | 0.302 | 0.002 (0.002) | 0.322 | 0.002 (0.002) | 0.269 | 0.0002 (0.002) | 0.915 | 0.003 (0.002) | 0.103 |
| Cancer has reoccurred | 0.273 (0.183) | 0.136 | 0.452 (0.182) | 0.014 | 0.316 (0.184) | 0.087 | 0.387 (0.183) | 0.035 | 17.483 (1.000) | <0.001 |
| Had surgery | -0.216 (0.137) | 0.115 | -0.095 (0.137) | 0.491 | 0.006 (0.137) | 0.967 | -0.334 (0.136) | 0.015 | -0.014 (0.137) | 0.917 |
| Had chemotherapy | 0.377 (0.110) | <0.001 | 0.322 (0.110) | 0.004 | 0.333 (0.110) | 0.003 | 0.550 (0.108) | <0.001 | 0.304 (0.110) | 0.006 |
| Had radiation | 0.309 (0.113) | 0.006 | 0.285 (0.113) | 0.012 | 0.279 (0.113) | 0.014 | 0.526 (0.110) | <0.001 | 0.232 (0.113) | 0.041 |
| The reference category for age is ‘35 up to 55 years old’.  The reference category for relationship status is ‘not in a relationship’ and ‘little or no support’ for partner support.  The reference category for type of cancer is Ovarian. | | | | | | | | | | |
